# Biological properties of *Elaeagnus rhamnoides* (L.) A. Nelson twig and leaf extracts

**DOI:** 10.1186/s12906-019-2564-y

**Published:** 2019-06-25

**Authors:** Bartosz Skalski, Bogdan Kontek, Bernadetta Lis, Beata Olas, Łukasz Grabarczyk, Anna Stochmal, Jerzy Żuchowski

**Affiliations:** 1 Department of General Biochemistry, Faculty of Biology and Environmental Protection, University of Łódź, Pomorska 141/3, 90-236 Łódź, Poland; 2 Department of Biochemistry, Institute of Soil Science and Plant Cultivation, State Research Institute, Czartoryskich 8, 24-100 Puławy, Poland; 3 Department of Neurology and Neurosurgery, Faculty of Medical Sciences, University of Warmia and Mazury, Warszawska 30, 10-082 Olsztyn, Poland

**Keywords:** Oxidative stress, *Elaeagnus rhamnoides* (L.) a. Nelson, Twig, Leaf, Berry, Phenolic compounds, Hemostasis

## Abstract

**Background:**

Sea buckthorn (*Elaeagnus rhamnoides* (L.) A. Nelson, SBT) is a valuable plant because of its medical and therapeutic potential. Different bioactive compounds in SBT berries are of special interest to various researchers. However, not only sea buckthorn berries, but also leaves of this plant (both fresh and dried) contain a lot of nutrients and bioactive compounds, including phenolic compounds. The present study was carried out in order to investigate antioxidant and anticoagulant properties of sea buckthorn twig and leaf extracts (0.5–50 μg/mL) by using various in vitro models. Moreover, the aim of present experiments was to compare the biological activity of SBT leaf extract and SBT twig extract with selected berry extracts (a rich source of phenolic compounds): SBT berry extract (flavonoids being the dominant components), a commercial extract from the berries of *Aronia melanocarpa* (Aronox**®**), and a grape seed extract.

**Methods:**

We determined the effect of plant extracts on the oxidative stress using selected markers of this process, i.e. the level of carbonyl groups in proteins. Additionally, we analysed the potential mechanism of modulation of hemostatic properties of human plasma (using selected coagulation times).

**Results:**

SBT twig and leaf extracts were observed to exhibit an antioxidant activity against two strong biological oxidants: hydrogen peroxide (H_2_O_2_) and H_2_O_2_/Fe (the donor of hydroxyl radicals), which induced human plasma lipid peroxidation and protein carbonylation. Both extracts also showed anticoagulant properties.

**Conclusions:**

Our present results have demonstrated that extracts from different parts of SBT, especially berries and twigs, in comparison to well-known berries (aronia and grape), may also be viewed as a good source of active substances – antioxidants for pharmacological or cosmetic applications. Moreover, it is very important from an economic point of view to know that there is a possibility of obtaining phenolic compounds not only from the berries or leaves, but also from twigs, which constitute a production waste.

## Background

Sea buckthorn (*Elaeagnus rhamnoides* (L.) A. Nelson, SBT) is an important plant because of its immense medical and therapeutic potential [1–4]. Different bioactive compounds in SBT berries are of special interest to various researchers [1, 5, 6]. However, not only sea buckthorn berries, but also leaves of this plant (both fresh and dried) contain large amounts of nutrients and bioactive compounds, including phenolic compounds [7]. Over the recent years, SBT leaf extracts have been scientifically investigated and various biological properties, i.e. radioprotective, anti-inflammatory and immunomodulatory, have been reported [1, 7, 8]. Results of Lee et al. [9] and Pichiah et al. [10] demonstrated that SBT leaves (used in the form of teas and extracts) possess anti-obesity properties. Recently, Sadowska et al. [11] have shown that not only SBT leaf extract, but also its twig extract, have anti-virulence action in vitro. However, lack of interest in the potential value of these extracts, especially SBT twig extract as the source of antioxidants and anticoagulants, is surely a significant hindrance for the development of alternative substances for prevention and treatment of cardiovascular diseases, which are frequently associated with oxidative stress and changes in hemostasis.

The aim of present experiments was to determinate the potential of SBT twig extract components and SBT leaf extract components for: (I) modulation of oxidative stress in human plasma treated with a strong biological oxidant: hydrogen peroxide (H_2_O_2_) and H_2_O_2_/Fe (the donor of hydroxyl radicals) (using selected markers of oxidative stress, i.e. the level of carbonyl groups in proteins); (II) modulation of hemostatic properties of human plasma (using selected coagulation times). It should be also emphasized that a novel aspect of our study focused on the comparison of biological activity of SBT leaf extract and SBT twig extract with selected berry extracts (rich in phenolic compounds): SBT berry extract (flavonoids were the dominant components [3, 4]), a commercial extract from the berries of *Aronia melanocarpa* (black chokeberry or aronia berry; Aronox**®**), and a grape seed extract, which displays not only antioxidative, but also anticoagulant and antiplatelet properties [2, 4, 12–14].

## Methods

### Reagents

Dimethylsulfoxide (DMSO), thiobarbituric acid (TBA), H_2_O_2_, and formic acid (LC-MS grade) were acquired from Sigma-Aldrich (St. Louis, MO., USA). Methanol (isocratic grade) and acetonitrile (LC-MS grade) were purchased from Merck (Darmstadt, Germany). All remaining reagents represented analytical grade and were provided by commercial suppliers.

A stock solution of *A. melanocarpa* berry extract (commercial product – Aronox**®** by Agropharm Ltd., Poland; batch No. 020/2007 k) was prepared in H_2_O at a concentration of 5 mg/mL, then kept frozen and subsequently used for experiments. The total content of phenolics in the phenolic-rich powder used in this study amounted to 309.6 mg/g of extract, including phenolic acids (isomers of chlorogenic acid) – 149.2 mg/g of extract, anthocyanins (anthocyanin glycosides: cyanidin 3-galactoside, cyanidin 3-glucoside, cyanidin 3-arabinoside, cyanidin 3-xyloside) – 110.7 mg/g, and flavonoids (quercetin glycosides) – 49.7 mg/g of extract. The HPLC determination of the phenolic-rich extract from *A. melanocarpa* berries had been previously described [12–14].

The grape seed extract was supplied by Bionorica (Germany) and was characterized by a total content of phenolics equalling 500 mg/g of extract [13]. A stock solution of grape seed extract was prepared in 50% DMSO.

### Plant material

Sea buckthorn berries, twigs and leaves were harvested from a horticultural farm in Sokółka, Podlaskie Voivodeship, Poland (53°24′N, 23°30′E), the greatest Polish producer of sea buckthorn fruits. The plant material was identified by Mr. Stanislaw Trzonkowski, the owner of the farm. A voucher specimens have been deported at the Institute of Soil Science and Plant Cultivation – Sate Research Institute, Pulawy, Poland (IUNG/HRH/2015/2).

### Chemical characteristics of the extract of phenolic compounds from sea buckthorn berries, twigs and leaf

Extracts from the fruit, leaves and twigs of sea buckthorn were prepared as previously described [3, 11]. Their composition was determined by reverse-phase UHPLC-MS/MS, using ACQUITY UPLC™ system (Waters, Milford, MA, USA), coupled with an ACQUITY TQD (Waters) triple quadrupole mass detector. Chromatographic separations were performed on an ACQUITY HSS C18 (100 × 2.1 mm, 1.8 µm; Waters) column (the fruit extract) and an ACQUITY BEH C18 (100 mm × 2.1 mm, 1.7 μm; Waters) column (leaf and twig extracts). Components of the extracts were identified on the basis of their MS and UV spectra, as well as literature data [15–17].

Stock solutions of the SBT berry extract, SBT twig extract and SBT leaf extract were made in 50% DMSO. The final concentration of DMSO in tested samples was lower than 0.05% and its effects were determined in all experiments.

### Plasma isolation

Fresh human plasma and blood were obtained from healthy and medication-free donors of a blood bank at a Medical Center (Lodz, Poland). Moreover, blood was obtained from non-smoking men and women (collected into CPD solution (citrate/phosphate/dextrose; 9:1; v/v blood/CPD) or CPDA solution (citrate/phosphate/dextrose/adenine; 8.5:1; v/v; blood/CPDA)). Our analysis of the blood samples was performed under the guidelines of the Helsinki Declaration for Human Research, and approved by the Committee on the Ethics of Research in Human Experimentation at the University of Lodz (resolution No. 3/KBBN-UŁ/II/2016). Plasma was incubated (15, 30 or 60 min, at 37 °C) with:

- SBT extracts at the final concentrations of 0.5–50 μg/mL
- SBT extracts at the final concentrations of 0.5–50 μg/mL plus 2 mM H_2_O_2_
- SBT extracts at the final concentrations of 0.5–50 μg/mL plus 4.7 mM H_2_O_2_/3.8 mM Fe_2_SO_4_/2.5 mM EDTA
- Aronia berry extract or grape seed extract at the final concentration of 50 μg/mL
- Aronia berry extract or grape seed extract at the final concentration of 50 μg/mL plus 2 mM H_2_O_2_
- Aronia berry extract or grape seed extract at the final concentration of 50 μg/mL plus 4.7 mM H_2_O_2_/3.8 mM Fe_2_SO_4_/2.5 mM EDTA.

The protein concentration, determined by measuring absorbance at 280 nm (in tested samples), was calculated according to the procedure of Whitaker and Granum [18].

### Markers of oxidative stress

#### Lipid peroxidation measurement

Lipid peroxidation was quantified by measuring the concentration of TBARS. Absorbance was measured at 535 nm (the SPECTROstar Nano Microplate Reader- BMG LABTECH Germany) [19, 20]. The TBARS concentration was calculated using the molar extinction coefficient (ε = 156,000 M^− 1^ cm^− 1^). More details were described in Skalski et al. [21].

#### Carbonyl group measurement

The detection of carbonyl groups in proteins was carried out according to Levine et al. [22] and Bartosz [20]. The carbonyl group concentration was calculated using a molar extinction coefficient (ε = 22,000 M^− 1^ cm^− 1^). The level of carbonyl groups was presented as nmol carbonyl groups/mg of protein. More details were described in Olas et al. [23].

#### Thiol group determination

The level of thiol group was measured spectrophotometrically (the SPECTROstar Nano Microplate Reader- BMG LABTECH Germany) by absorbance at 412 nm with Ellman’s reagent: 5,5′-dithio-bis-(2-nitrobenzoic acid). The level of thiol groups was expressed as nmol thiol groups/mg of plasma protein [24, 25]. More details were described in Olas et al. [23].

### Parameters of hemostasis

#### The measurement of prothrombin time (PT)

Human plasma was incubated at 37 °C on a block heater. After incubation, the cuvette was transferred to measuring holes. Then 100 μL of Dia-PT liquid (commercial preparation) was added. The PT was determined coagulometrically using Optic Coagulation Analyser model K-3002 [26].

#### The measurement of thrombin time (TT)

Human plasma was added to a coagulometric cuvette and incubated at 37 °C on a block heater. Then the cuvette was transferred to measuring holes and 100 μL of thrombin (final concentration - 5 U/mL) was added. The TT was determined coagulometrically using Optic Coagulation Analyser model K-3002 [26].

#### The measurement of activated partial thromboplastin time (APTT)

Human plasma was added to a coagulometric cuvette. Then the incubation was conducted at 37 °C on a block heater with 50 μL of Dia-PTT liquid (commercial preparation). The cuvette was transferred to measuring holes. Then 50 μL of 25 mM CaCl_2_ was added. The APTT was determined coagulometrically (Optic Coagulation Analyser model K-3002) [26].

### Data analysis

Several tests were used to carry out statistical analysis. All the values in this study were expressed as mean ± SD. Obtained results were analysed under the account of normality with Shapiro-Wilk test and equality of variance with Levine test. Statistical significance of differences among experimental variants was assessed by ANOVA (the significance level was *p* < 0.05), followed by Tukey multiple comparison test or Kruskal-Wallis test.

## Results

The UHPLC-MS analyses demonstrated that different glycosides of isorhamnetin and quercetin (with isorhamnetin 3-*O*-Hex-dHex; isorhamnetin 3-*O*-Hex, and isorhamnetin 3-*O*-Hex-7-*O*-dHex as dominant compounds) were main constituents of the phenolic extract of sea buckthorn berries and their total amount, expressed as isorhamnetin 3-O-β-glucosyl-(1 → 2)-β-galactoside equivalent (214.04 mg/g). Other phenolic compounds were difficult to identify and most of them were present in small amounts. Their total content was 28.65 mg/g of the extract (expressed as isorhamnetin 3-O-β-glucosyl-(1 → 2)-β-galactoside equivalent) [3]. Ellagitannins (259.6 ± 3.1 mg/g) were identified as principal phenolic constituents of the SBT leaf extract. Flavonoids (74.7 ± 0.7 mg/g) were represented by glycosides of isorhamnetin (the dominant aglycone), quercetin, and kaempferol. The SBT twig extract consisted mainly of B –type proanthocyanidins and catechin (the total content 597.1 ± 10.2 mg/g). More details can be found in the original literature [3, 11]. The total content of phenolics in SBT berry extract, SBT twig extract and SBT leaf extract is demonstrated in Table 1.

**Table 1:** Total content of phenolics in the extracts used in this experiment [3, 4, 11–14, 23]

Tested extract	Total content of phenolics
Aronia berry extract (commercial product, Aronox®, by Agropharm Ltd. Poland)	309.8 mg/g of extract
Grape seed extract (by Bionorica, Germany)	500 mg/g of extract
SBT berry extract (phenolic extract)	242.7 mg/g of fraction
SBT leaf extract (butanolic extract)	341.5 mg/g of extract
SBT twig extract (butanolic extract)	621.2 mg/g of extract

**Fig. 1:**
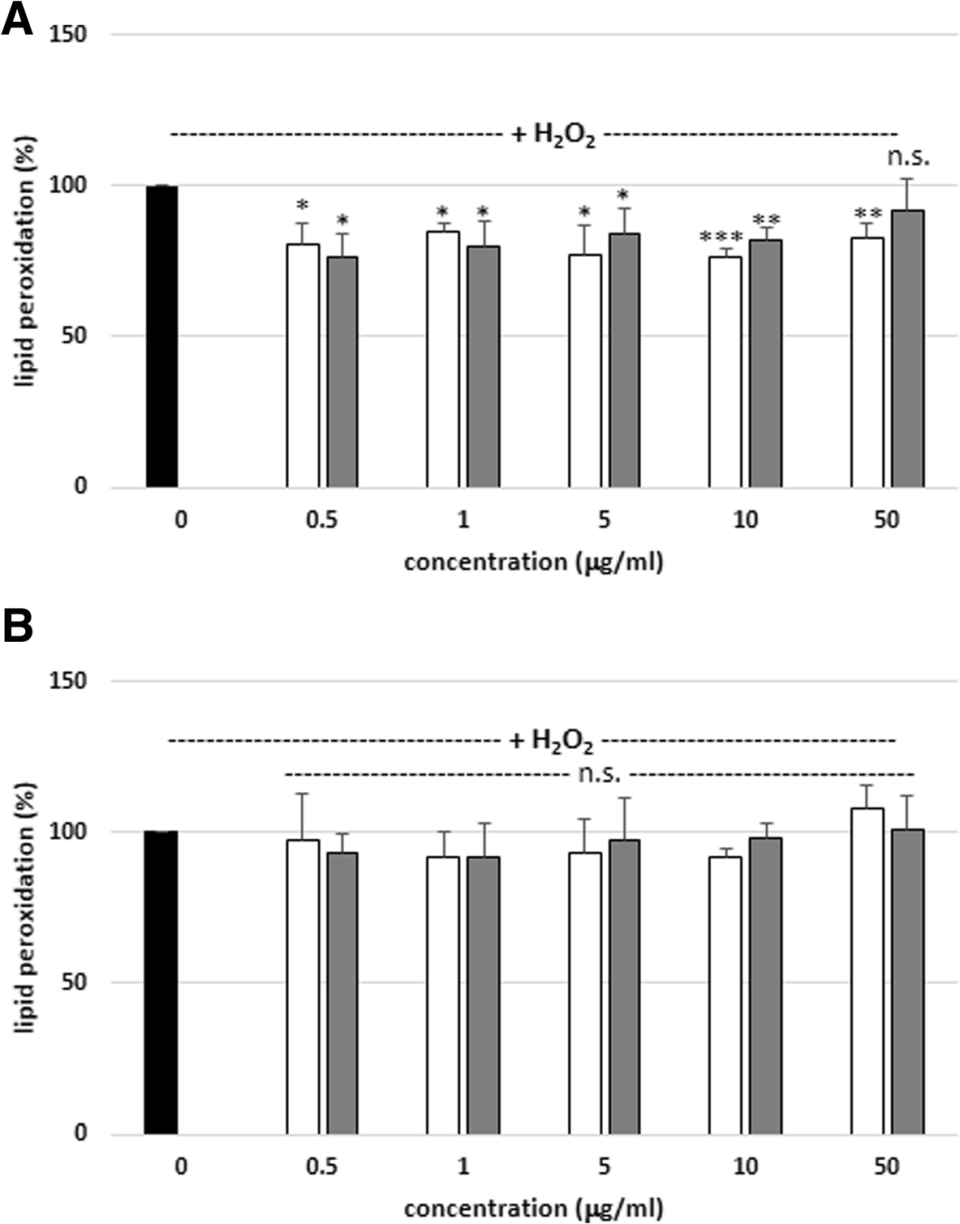
Effects of SBT twig and leaf extracts (0.5–50 μg/mL; 15 min (**a**) and 60 min (**b**)) on plasma lipid peroxidation induced by H_2_O_2_. In these experiments, the TBARS level (marker of lipid peroxidation) in control samples (plasma treated with only H_2_O_2_) was 0.254 ± 0.046 nmol/mL of plasma. Data represent means ± SD of 5–10. The effect of five different concentrations of two tested extracts (0.5, 1, 5, 10 and 50 μg/mL; for 15 min) was statistically significant (*p < 0.05, **p < 0.005; ***p < 0.001) in comparison to control. The effect of five different concentrations of two tested extracts (0.5, 1, 5, 10 and 50 μg/mL; for 60 min) was not statistically significant (*p* > 0.05 (n.s.)) in comparison to control. The effects were not statistically significant: SBT twig extract-treated plasma vs. SBT leaf extract-treated plasma (*p* > 0.05 (n.s.); for all tested concentrations- 0.5 - 50 μg/mL; for 15 and 60 min). black diagram – control, white diagram – twig, grey diagram - leaf

**Fig. 2:**
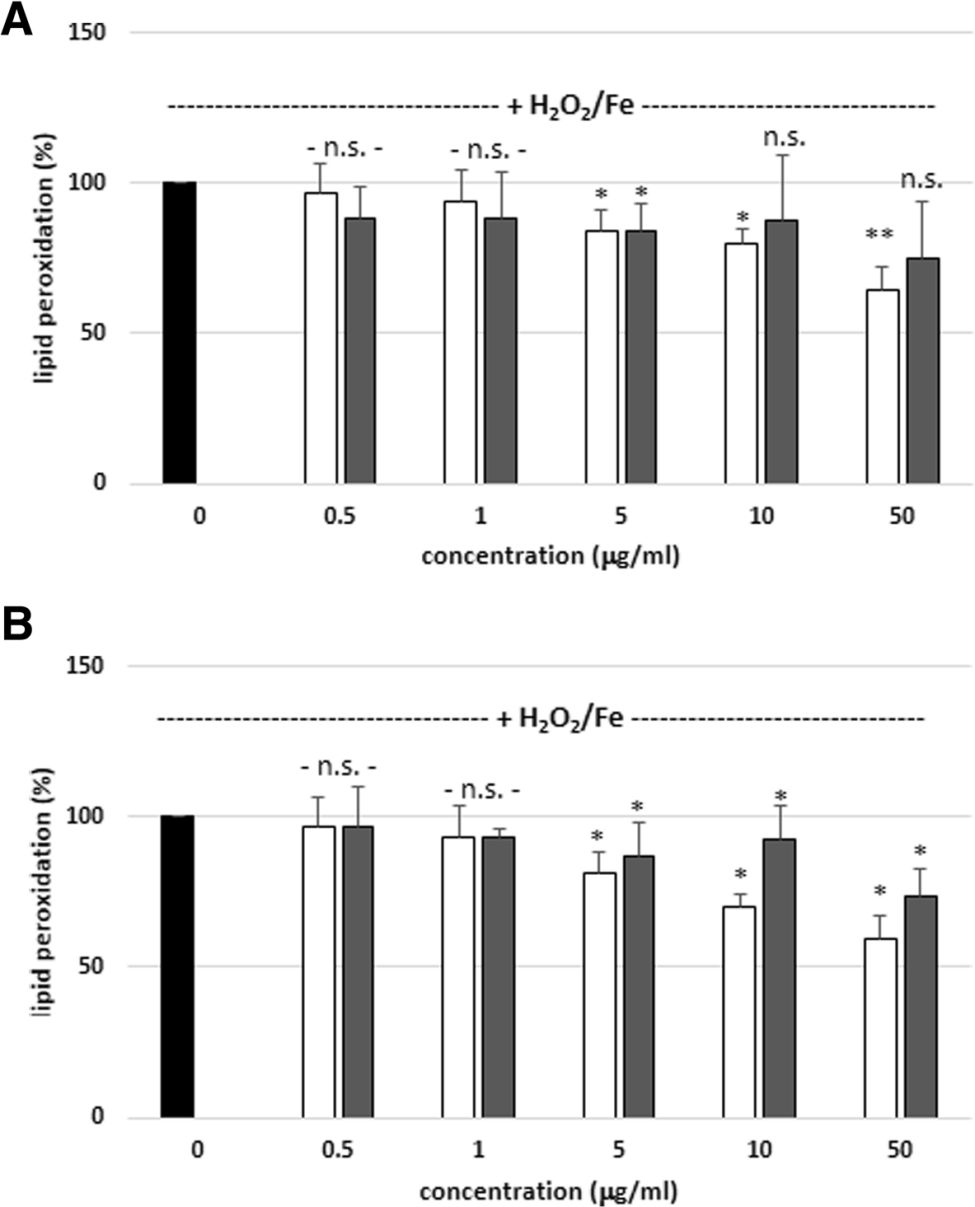
Effects of SBT twig and leaf extracts (0.5–50 μg/mL; 15 min (**a**) and 60 min (**b**)) on plasma lipid peroxidation induced by H_2_O_2_/Fe. In these experiments, the TBARS level (marker of lipid peroxidation) in control samples (plasma treated with only H_2_O_2_/Fe) was 0.341 ± 0.078 nmol/mL of plasma. Data represent means ± SD of 5–10. The effect of two different concentrations of two tested extracts (0.5 and 1 μg/mL; for 15 and 60 min) was not statistically significant (*p* > 0.05 (n.s.)) in comparison to control. The effect of two different concentrations of SBT leaf extract (10 and 50 μg/mL; for 15 min) was not statistically significant (*p* > 0.05 (n.s.)) in comparison to control. The effect of three different concentrations of SBT twig extract (5, 10 and 50 μg/mL; for 15 min) was statistically significant (*p < 0.05, **p < 0.005) in comparison to control. The effect of one concentration of SBT leaf extract (5 μg/mL; for 15 min) was statistically significant (*p < 0.05) in comparison to control. The effect of three different concentrations of two tested extracts (5, 10 and 50 μg/mL; for 60 min) was statistically significant (*p < 0.05) in comparison to control. The effects were not statistically significant: SBT twig extract-treated plasma vs. SBT leaf extract-treated plasma (for 15 min: *p* > 0.05 (n.s.), for all tested concentrations- 0.5 - 50 μg/mL); for 60 min (*p* > 0.05 (n.s.), for tested concentrations: 0.5, 1 and 10 μg/mL). The effects were statistically significant: SBT twig extract-treated plasma vs. SBT leaf extract-treated plasma, (for 60 min, *p* < 0.05 for tested concentrations: 10 and 50 μg/mL). black diagram – control, white diagram – twig, grey diagram - leaf

Antioxidant properties of plant extracts cannot be evaluated by a single method, due to the complex nature of phytochemicals. Therefore, in the present study antioxidant activity of tested plant extracts was evaluated on the basis of their influence on levels of lipid peroxidation, carbonyl groups and thiol groups in human plasma. The antioxidant activity of SBT twig and leaf extracts (at the concentrations: 0.5–50 μg/mL; incubation time: 15 and 60 min) were studied in vitro. As demonstrated in Fig. 1a, two tested extracts inhibited lipid peroxidation in human plasma treated with H_2_O_2_, but this inhibition was not concentration-dependent for 15 min of incubation time. However, we observed that the two tested extracts (at all concentrations) did not change plasma lipid peroxidation (induced by H_2_O_2_) when longer incubation time (60 min) was applied (Fig. 1b). On the other hand, SBT twig extract (at doses: 5, 10 and 50 μg/mL, for 15 and 60 min of incubation time) reduced the level of plasma lipid peroxidation induced by H_2_O_2_/Fe; additionally, activity of the extract was concentration-dependent (Fig. 2a and b). SBT leaf extract revealed antioxidant properties at the same concentrations, but only for 60 min of incubation time (Fig. 2b). Moreover, in this model (with H_2_O_2_/Fe as the inducer of oxidative stress), SBT twig extract (at the highest concentration – 50 μg/mL; 60 min) demonstrated stronger antioxidant properties than SBT leaf extract (at the same concentration) (Tab. 2). SBT twig extract reduced lipid peroxidation by about 40%, and SBT leaf extract by about 30% (Tab. 2).

**Table 2:** Comparison of antioxidant properties of SBT twig and leaf extracts with properties of selected berry extracts (50 μg/mL; 15 and 60 min) in human plasma. Data represent means ± SD of 5–12. The level of marker of oxidative stress in control sample (plasma treated with H_2_O_2_ or H_2_O_2_/Fe) was expressed as 100%

% of lipid peroxidation induced by H_2_O_2_ (incubation time – 15 min)	
Control	100
SBT leaf extract (A)	91.9 ± 10.5
SBT twig extract (B)	82.5 ± 4.9; B vs A (*p* > 0.05 (n.s.))
Aronia berry extract (C)	83.1 ± 10.4; C vs A (*p* > 0.05 (n.s.)); C vs B (*p* > 0.05 (n.s.))
Grape seed extract (D)	79.4 ± 9.9; D vs A (*p* > 0.05 (n.s.)); D vs B (p > 0.05 (n.s.))
SBT berry extract (E)	60.3 ± 12.1; E vs A (*p* < 0.01); E vs B (*p* < 0.01)
% of lipid peroxidation induced by H_2_O_2_ (incubation time – 60 min)	
Control	100
SBT leaf extract (A)	107.9 ± 8.0
SBT twig extract (B)	101.1 ± 10.9; B vs A (*p* > 0.05 (n.s.))
Aronia berry extract (C)	91.0 ± 3.2; C vs A (*p* > 0.05 (n.s.)); C vs B (*p* > 0.05 (n.s.))
Grape seed extract (D)	97.2 ± 5.5; D vs A (*p* > 0.05 (n.s.)); D vs B (*p* > 0.05 (n.s.))
SBT berry extract (E)	39.4 ± 7.7; E vs A (*p* < 0.001); E vs B (*p* < 0.001)
% of lipid peroxidation induced by H_2_O_2_/Fe (incubation time – 15 min)	
Control	100
SBT leaf extract (A)	74.6 ± 19.1
SBT twig extract (B)	64.5 ± 15.6; B vs A (*p* > 0.05 (n.s.))
Aronia berry extract (C)	61.4 ± 11.2; C vs A (*p* > 0.05 (n.s.)); C vs B (*p* > 0.05 (n.s.))
Grape seed extract (D)	73.5 ± 8.8; D vs A (*p* > 0.05 (n.s.)); D vs B (*p* > 0.05 (n.s.))
SBT berry extract (E)	30.4 ± 9.7; E vs A (*p* < 0.01); E vs B (*p* < 0.01)
% of lipid peroxidation induced by H_2_O_2_/Fe (incubation time – 60 min)	
Control	100
SBT leaf extract (A)	73.2 ± 9.7
SBT twig extract (B)	59.7 ± 7.5; B vs A (*p* < 0.05)
Aronia berry extract (C)	89.3 ± 9.5; C vs A (*p* < 0.05); C vs B (*p* < 0.05)
Grape seed extract (D)	85.0 ± 8.8; D vs A (*p* < 0.05); D vs B (*p* < 0.05)
SBT berry extract (E)	59.2 ± 9.5; E vs A (*p* < 0.05); E vs B (*p* > 0.05 (n.s.))
% of protein carbonylation induced by H_2_O_2_ (incubation time – 15 min)	
Control	100
SBT leaf extract (A)	95.4 ± 13.9
SBT twig extract (B)	80.8 ± 26.7; B vs A (*p* > 0.05 (n.s.))
Aronia berry extract (C)	99.7 ± 17.3; C vs A (*p* > 0.05 (n.s.)); C vs B (*p* > 0.05 (n.s.))
Grape seed extract (D)	95.4 ± 11.4; D vs A (*p* > 0.05 (n.s.)); D vs B (*p* > 0.05 (n.s.))
SBT berry extract (E)	66.4 ± 10.3; E vs A (*p* < 0.01); E vs B (*p* > 0.05 (n.s.))
% of protein carbonylation induced by H_2_O_2_ (incubation time – 60 min)	
Control	100
SBT leaf extract (A)	55.6 ± 19.7
SBT twig extract (B)	56.4 ± 18.9; B vs A (*p* > 0.05 (n.s.))
Aronia berry extract (C)	92.4 ± 11.4; C vs A (*p* < 0.01; C vs B (*p* < 0.01)
Grape seed extract (D)	74.3 ± 10.5; D vs A (*p* < 0.05); D vs B (*p* < 0.05)
SBT berry extract (E)	61.4 ± 9.7; E vs A (*p* > 0.05 (n.s.)); E vs B (*p* > 0.05 (n.s.))
% of protein carbonylation induced by H_2_O_2_/Fe (incubation time – 15 min)	
Control	100
SBT leaf extract (A)	96.4 ± 8.1
SBT twig extract (B)	97.7 ± 5.1; B vs A (*p* > 0.05 (n.s.))
Aronia berry extract (C)	99.0 ± 15.5; C vs A (*p* > 0.05 (n.s.)); C vs B (*p* > 0.05 (n.s.))
Grape seed extract (D)	96.7 ± 12.4 D vs A (*p* > 0.05 (n.s.)); D vs B (*p* > 0.05 (n.s.))
SBT berry extract (E)	79.4 ± 10.2; E vs A (*p* < 0.02); E vs B (*p* < 0.02)
% of protein carbonylation induced by H_2_O_2_/Fe (incubation time – 60 min)	
Control	100
SBT leaf extract (A)	53.3 ± 21.4
SBT twig extract (B)	75.6 ± 18.4; B vs A (*p* < 0.05)
Aronia berry extract (C)	82.6 ± 15.1; C vs A (*p* < 0.05); C vs B (*p* > 0.05 (n.s.))
Grape seed extract (D)	80.5 ± 17.3; D vs A (*p* < 0.05); D vs B (*p* > 0.05 (n.s.))
SBT berry extract (E)	69.4 ± 12.3; E vs A (*p* > 0.05 (n.s.)); E vs B (*p* > 0.05 (n.s.))

**Fig. 3:**
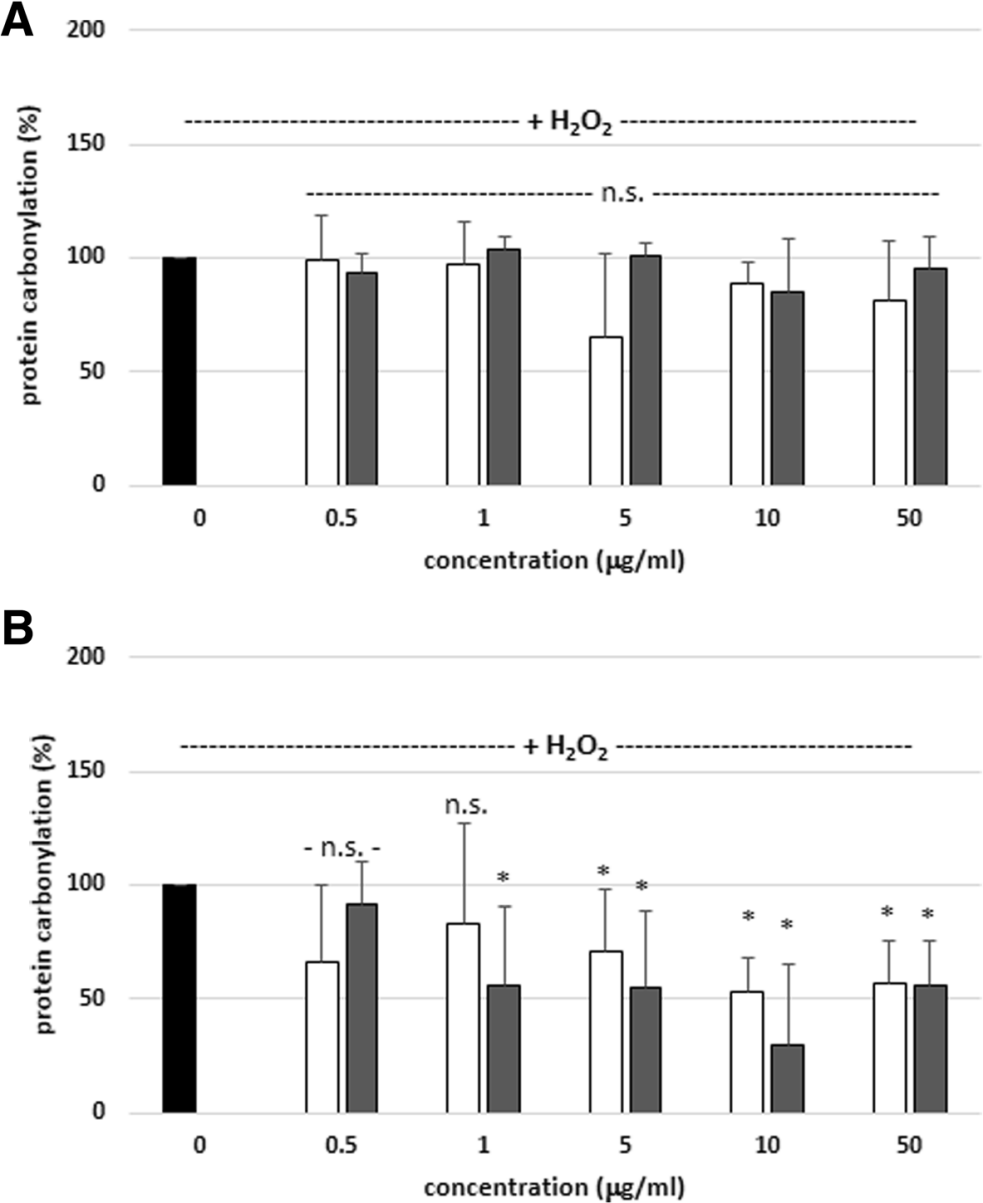
Effects of SBT twig and leaf extracts (0.5–50 μg/mL; 15 min (**a**) and 60 min (**b**)) on plasma protein carbonylation induced by H_2_O_2_. In these experiments the carbonyl group level (marker of protein oxidation) in control samples (plasma treated only with H_2_O_2_) was 17.1 ± 4.3 nmol/mg of plasma protein. Data represent means ± SD of 6–12. The effect of five different concentrations of two tested extracts (0.5, 1, 5, 10 and 50 μg/mL; for 15 min) was not statistically significant (*p* > 0.05 (n.s.)) in comparison to control. The effect of two different concentrations of SBT twig extracts (0.5 and 1 μg/mL; for 60 min) was not statistically significant (*p* > 0.05 (n.s.)) in comparison to control. The effect of one concentration of SBT leaf extract (0.5 μg/mL; for 60 min) was not statistically significant (*p* > 0.05 (n.s.)) in comparison to control. The effect of three different concentrations of SBT twig extracts (5, 10 and 50 μg/mL; for 60 min) was statistically significant (*p < 0.05), in comparison to control. The effect of four different concentrations of SBT leaf extracts (1, 5, 10 and 50 μg/mL; for 60 min) was statistically significant (*p < 0.05), in comparison to control. The effects were not statistically significant: SBT twig extract-treated plasma vs. SBT leaf extract-treated plasma (for 15 and 60 min, *p* > 0.05 (n.s.); for all tested concentrations- 0.5 - 50 μg/mL). black diagram – control, white diagram – twig, grey diagram - leaf

**Fig. 4:**
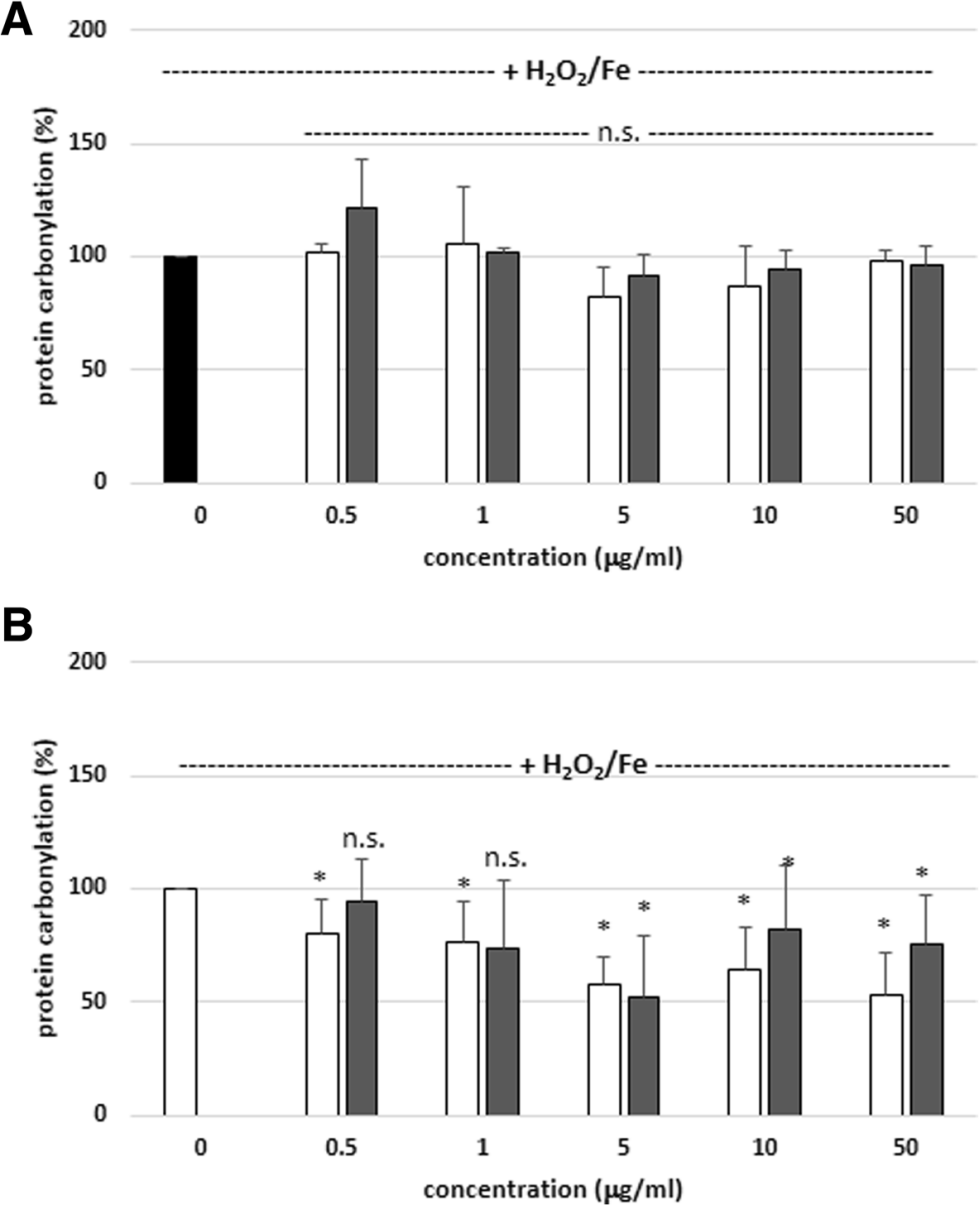
Effects of SBT twig and leaf extracts (0.5–50 μg/mL; 15 min (**a**) and 60 min (**b**)) on plasma protein carbonylation induced by H_2_O_2_/Fe. In these experiments the carbonyl group level (marker of protein oxidation) in control samples (plasma treated only with H_2_O_2_/Fe) was 30.4 ± 5.1 nmol/mg of plasma protein. Data represent means ± SD of 6–12. The effect of five different concentrations of two tested extracts (0.5, 1, 5, 10 and 50 μg/mL; for 15 min) was not statistically significant (*p* > 0.05 (n.s.)) in comparison to control. The effect of five different concentrations of SBT twig extract (0.5, 1, 5, 10 and 50 μg/mL; for 60 min) was statistically significant (*p < 0.05) in comparison to control. The effect of three different concentrations of SBT leaf extract (5, 10 and 50 μg/mL; for 60 min) was statistically significant (*p < 0.05) in comparison to control. The effect of two different concentrations (0.5 and 1 μg/mL; for 60 min) was not statistically significant (*p* > 0.05 (n.s.)), in comparison to control. The effects were not statistically significant: SBT twig extract-treated plasma vs. SBT leaf extract-treated plasma (for 15 min, *p* > 0.05 (n.s.); for all tested concentrations- 0.5 - 10 μg/mL). The effects were not statistically significant: SBT twig extract-treated plasma vs. SBT leaf extract-treated plasma (for 60 min, *p* > 0.05 (n.s.); for three tested concentrations- 0.5 - 5 μg/mL). The effects were statistically significant: SBT twig extract-treated plasma vs. SBT leaf extract-treated plasma (for 60 min, *p* < 0.05; for two tested concentrations- 10 and 50 μg/mL). black diagram – control, white diagram – twig, grey diagram - leaf

Another set of experiments focused on plasma protein carbonylation levels; the tested extracts had no effect on this process during short incubation – 15 min (Figs. 3a and 4a). Nevertheless,, the protein carbonylation (induced by H_2_O_2_ and H_2_O_2_/Fe) was reduced in the presence of SBT twig and leaf extracts, when a longer incubation time was applied – 60 min (Fig. 3b and 4b). In addition, SBT twig extract (at 50 μg/mL, incubation time – 60 min) had stronger inhibitory effect on plasma protein carbonylation (induced by H_2_O_2_/Fe) than SBT leaf extract (at 50 μg/mL, incubation time – 60 min); i.e. inhibition of this process was only about 53% for SBT leaf extract (50 μg/mL), and about 76% for SBT twig extract (50 μg/mL) (Fig. 4b, Table 2).

**Fig. 5:**
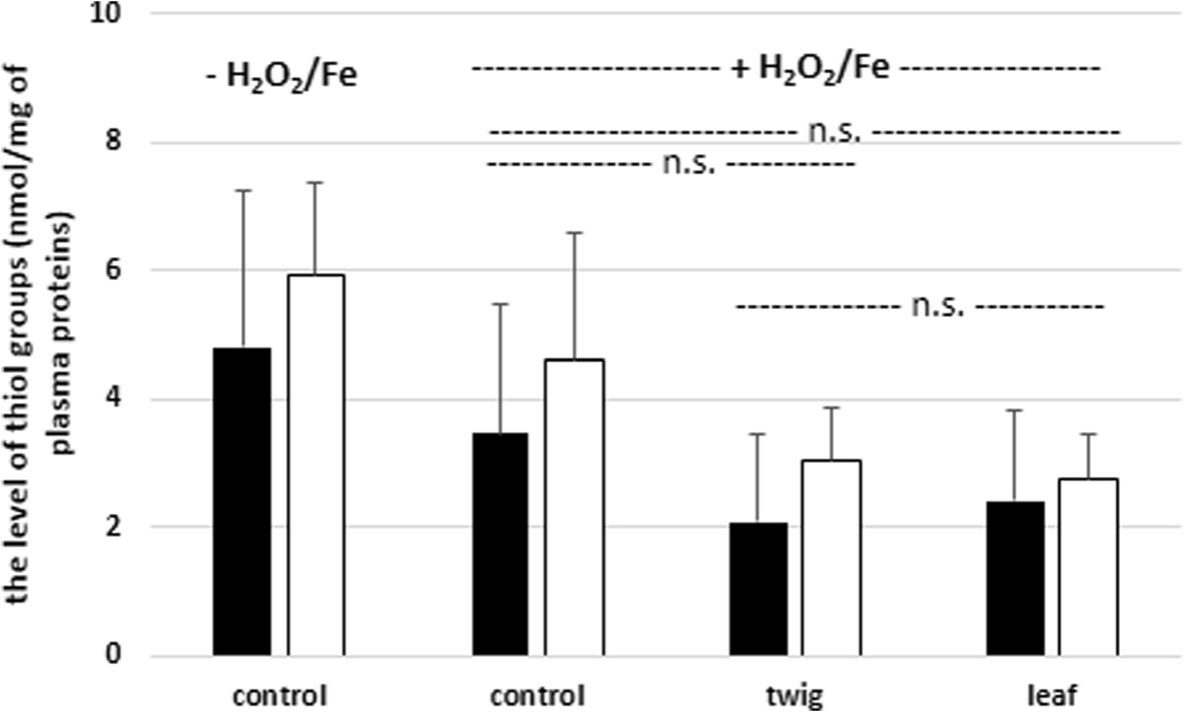
Effects of SBT twig and leaf extracts (50 μg/mL; 15 min and 60 min) on oxidation of plasma protein thiols induced by H_2_O_2_/Fe. Data represent means ± SD of 6–9; *p* > 0.05 (n.s.). Control negative refers to plasma not treated with H_2_O_2_/Fe, whereas control positive to plasma treated with H_2_O_2_/Fe. black diagram – 15 min, white diagram – 60 min

Analysis of the effect of tested extracts (50 μg/mL) on oxidation of plasma protein thiols demonstrated that SBT twig extract and leaf extract did not affect the said process (Fig. 5).

Moreover, we have demonstrated differences in antioxidant activity between SBT leaf or twig extract and selected berry extracts, i.e. SBT berry extract (butanolic extract). Table 2 shows comparative effects of SBT twig extract, SBT leaf extract and berry extracts, including SBT berry extract, aronia berry extract and grape seed extract (50 μg/mL) on the level of biomarkers of oxidative stress in human plasma. We observed that SBT berry extract had stronger antioxidant properties (especially for the inhibition of lipid peroxidation) than SBT twig and leaf extracts (Tab. 2), i.e. the inhibition of lipid peroxidation (induced by H_2_O_2_/Fe, incubation time – 15 min) reached about 70% (for SBT berry extract), about 25% (for SBT leaf extract) and about 35% (for SBT twig extract). However, antioxidant properties of SBT twig and leaf extracts were very often similar, like for aronia berry extract and grape seed extract (Tab. 2).

As shown in Table 3, SBT twig and leaf extracts (at the highest test concentration - 50 μg/mL; incubation time – 30 min) significantly prolonged the APTT time. We demonstrated that SBT twig extract had stronger activities than leaf extract. In addition, SBT twig extract had stronger anticoagulant activity than berry extracts (SBT berry extract, aronia berry extract and grape seed extract (Tab. 3). However, SBT twig and leaf extracts did not change the TT and the PT (data are not presented).

**Table 3:** Comparison of anticoagulant properties of SBT twig and leaf extracts with properties of selected berry extracts (50 μg/mL; 30 min). Data represent means ± SD of 12–30

APTT (s)	
Control (A)	42.3 ± 4.1
SBT leaf extract (B)	46.4 ± 4.5; B vs A (*p* < 0.05)
SBT twig extract (C)	51.9 ± 3.2; C vs A (*p* < 0.05); C vs B (*p* < 0.01)
Aronia berry extract (D)	45.3 ± 3.2; D vs A (*p* < 0.05); D vs C (*p* < 0.01)
Grape seed extract (E)	46.1 ± 2.9; E vs A (*p* < 0.05); E vs B (*p* < 0.02)
SBT berry extract (F)	40.2 ± 3.4; F vs A (*p* > 0.05 (n.s.)); F vs B (*p* < 0.001)

## Discussion

Sea buckthorn is a wild plant that has been used for centuries as a traditional medicine for treating different diseases. Over the last two decades, researchers have demonstrated a correlation between chemical composition and biological activity of SBT [3, 4, 27]. Researchers have also frequently correlated the SBT action with compounds present in its extracts, especially those from berries and leaves, however, less data is available regarding compounds from SBT twigs. Results obtained by different researchers have indicated that antioxidant activities of phenolic compounds from SBT berries and leaves may be partly responsible for the beneficial effects of these compounds on human health [28–30]. Our earlier results showed that a butanolic extract from SBT berries (rich in flavonoids) exhibits antioxidant and anti-platelet properties [3, 4]. Moreover, Tian et al. [31] analysed the chemical content of extracts from berries and leaves of 13 berries and leaves of various berry plants, including sea buckthorn. They observed that sea buckthorn leaves are the richest source of phenolics (7856 mg/100 g f.w.) with ellagitannins being the dominant compound class.

It is vital to note that SBT berries and leaves show no cytotoxicity or adverse effects upon oral administration [32–34]. Moreover, there is no report concerning the toxicity of SBT phenolic compounds. In addition, the range of tested concentrations of SBT leaf extract, SBT twig extract and selected berry extracts (0.5–50 μg/mL) in human plasma in our experiments can be achieved by way of oral supplementation with phenolics [35, 36].

An interesting aspect of beneficial effects of extracts from different parts of SBT on human health is its protective actions on the cardiovascular system [3, 4, 37]. The effect of various parts of SBT, especially twigs and leaves, on different components of hemostasis, including plasma, which plays a role in cardiovascular system efficiency, has not been studied yet. Thus, the main objective of our in vitro experiments was to examine the antioxidant and anticoagulant activities of SBT twig and leaf extracts in human plasma.

In the present study, the addition of H_2_O_2_ or H_2_O_2_/Fe to human plasma resulted in a significant increase in the level of different tested oxidative stress biomarkers. Two oxidative agents, namely (1) H_2_O_2_ and (2) H_2_O_2_/Fe were used, because Fe concentration in isolated plasma is low, similarly to the level of oxidative stress parameters (i.e. the level of TBARS and carbonyl groups in proteins), which is also low. However, when Fe is added to isolated human plasma, higher level of these markers can be noted. Moreover, some researchers indicate that certain complexes of iron ions (i.e. EDTA) may also react with hydrogen peroxide to form hydroxyl radicals [37, 38]. The results of our experiments indicate that SBT leaf extract exhibited an inhibitory action on H_2_O_2_ and H_2_O_2_/Fe – induced lipid peroxidation and protein carbonylation in human plasma in vitro. These results are consistent with other studies on the role of SBT leaf extract in protecting against oxidative stress [39, 40]. However, it is known that the nature and polarity of solvent may decide about biological activity of phenolic extracts from different parts of plants, i.e. Upadhyay et al. [8] have used two different leaf extracts: aqueous extract (total phenolics: 40.49 ± 2.10 mg gallic acid equivalents/g dry leaf) and hydroalcoholic extract (total phenolics: 56.28 ± 2.30 mg gallic acid equivalents/g dry leaf). They have found SBT leaf extracts to have not only antioxidant, but also cytoprotective and antibacterial effects. Both aqueous and hydroalcoholic extracts of SBT leaves (at concentration of 250 μg/mL) exhibited potent antioxidant activity when analysed by 2,2′-diphenyl-1-picrylhydrazyl (DPPH), 2,2′-azino-bis (3-ethylbenzothiazoline-6-sulfoni acid) diammonium salt (ABTS) and Ferric Reducing Antioxidant Power (FRAP). However, our present results demonstrate that tested SBT leaf extract (butanolic extract), even at low concentrations (0.5–50 μg/mL), could be used as a natural source of antioxidants, i.e. inhibition of plasma protein carbonylation (induced by H_2_O_2_) levelled about 45% (for the concentration of 50 μg/mL, incubation time – 60 min). Results obtained by Khan et al. [40] have shown that SBT leaf extract ameliorates the gamma radiation mediated DNA damage and hepatic alterations. For this in vivo experiment, Swiss albino mice have been administered SBT (30 mg/kg body weight) for 15 consecutive days before exposing them to a single dose of 5 Gy of beta radiation. Maheshwari et al. [39] also demonstrated that the phenolic-rich fraction of SBT leaves has a potent antioxidant activity, prevents oxidative damage to proteins and lipids, and affords significant protection against CCl_4_-stimulated oxidative liver damage in Sprague Dawley rats. In addition, results of Bala et al. [41] indicate that standardized leaf extract from sea buckthorn (administered 12 mg/kg body weight, before irradiation) normalized brain superoxide dismutase and catalase in rats. Cho et al. [42] showed that SBT leaf extracts protect neuronal PC-12 cells from oxidative stress in vitro. Moreover, SBT leaf extract inhibits rapid proliferation of rat C6 glioma cells, possibly by inducing early events of apoptosis [43].

Few phenolic constituents of the investigated extracts can be directly absorbed in the GI tract. During their passage through the GI tract, flavonol glycosides are usually hydrolysed by microbial and intestinal enzymes. Flavonol aglycons are partly decomposed by intestinal microbes (and products of the decomposition can be absorbed by intestines), partly absorbed by intestines; the absorbed flavonol glycosides occur in the circulation system mainly as sulfates, glucuronides or diglucuronides. Catechin can be partly absorbed in the GI tract (and most the absorbed catechin is further sulfated or glucuronized), partly decomposed by intestinal microbiota (and the decomposition products also can be absorbed). It seems oligomeric proanthocyanidins cannot be directly absorbed, but products of their microbial decomposition (mainly different phenolic acids) are absorbed into the circulatory system.

Ellagitannins (from pomegranates, raspberries, strawberries, walnuts) are not directly absorbed, but products of their microbial decomposition (so called urolithins, as well as ellagic acid) can be absorbed, and occur it the circulation system mainly as sulfates or glucuronides [44].

For the first time, our findings have demonstrated antioxidant properties of SBT twig extract (the butanolic extract) in an experimental system of isolated human plasma. The tested extract significantly reduced the action on H_2_O_2_ and H_2_O_2_/Fe – induced oxidation in human plasma in vitro. However, our earlier results have shown that not only the phenolic fractions, but also the non-polar fractions (rich in triterpenes and acylated triterpenes) from sea buckthorn twigs and leaf had antioxidant and anticoagulant properties [45].

Human plasma was used in our in vitro experiments because it is an important component of hemostasis. Changes in hemostasis are often correlated with oxidative stress, and oxidative stress has been implicated in development of cardiovascular diseases. A novel finding of this study is that SBT twig and leaf extracts (at the highest tested concentration – 50 μg/mL), similarly to aronia berry extract or grape seed extract, change coagulation properties of human plasma in vitro. They prolong the clotting time – APTT, which is a measure of the efficiency of intrinsic mechanism of activation of prothrombin, without blood platelets. Thus, the obtained results indicate anticoagulant activities of SBT twig and leaf extracts in model system in vitro.

We suppose that the differences in chemical profiles of tested extracts, especially total concentration of phenolic compounds, may explain differences in their biological activities (antioxidant and anticoagulant properties), i.e. they may explain the stronger action of SBT twig extract (than SBT leaf extract), in which proanthocyanidins exhibit high concentration (about 580 mg/g). We suppose that these compounds may act not only as main antioxidants in this extract, but also as compounds with anticoagulant activity. Our previous experiments have also demonstrated strong antioxidant potential of the phenolic fractions from sea buckthorn twigs, which can be attributed to a high content of catechin and proanthocyanidins [45]. Chong et al. [46] suggest that anthocyanidins, procyanidins, flavonols and phenolic acids may have the greatest beneficial impact on cardiovascular disorders. Other authors have also demonstrated that supplementation with commercial product made from aronia berries (Aronox®) results in improved clotting and fibrinolysis in patients with metabolic syndrome, and has been shown to modify hemostasis in in vitro [47]. Our present results are consistent with other studies concerning the anticoagulant properties of aronia berry extract, a known source of anthocyanins (about 110 mg/g). On the other hand, the non-polar fraction from sea buckthorn twigs (rich in triterpenoids and acylated triterpenoids) had a greater impact on coagulation system then the phenolic fraction [45].

## Conclusion

Extracts from different parts of SBT, especially berries and twigs, in comparison to well-known berries (aronia and grape) may be also a good source of active substances – anticoagulants and antioxidants for pharmacological or cosmetic applications. Moreover, it is very important from an economic point of view there is a possibility of obtaining phenolic compounds not only from berries or leaves, but also from twigs, which constitute a production waste.

## Data Availability

All data are presented in the manuscript. Data sets used and/or analysed in this study are available from the corresponding author on reasonable request.

## Abbreviations

APTT: Activated partial thromboplastin time
DMSO: Dimethylsulfoxide
H_2_O_2_: Hydrogen peroxide
PT: Prothrombin time
SBT: Sea buckthorn
TBA: Thiobarbituric acid
TT: Thrombin time
